# Supplementary figures and images for: RNA-Protein Interaction Analysis of SARS-CoV-2 5′ and 3′ Untranslated Regions Reveals a Role of Lysosome-Associated Membrane Protein-2a during Viral Infection
Source: mSystems. 2021 Jul 13;6(4):e00643-21. doi: 10.1128/mSystems.00643-21 (PMC8407388; doi:10.1128/mSystems.00643-21)

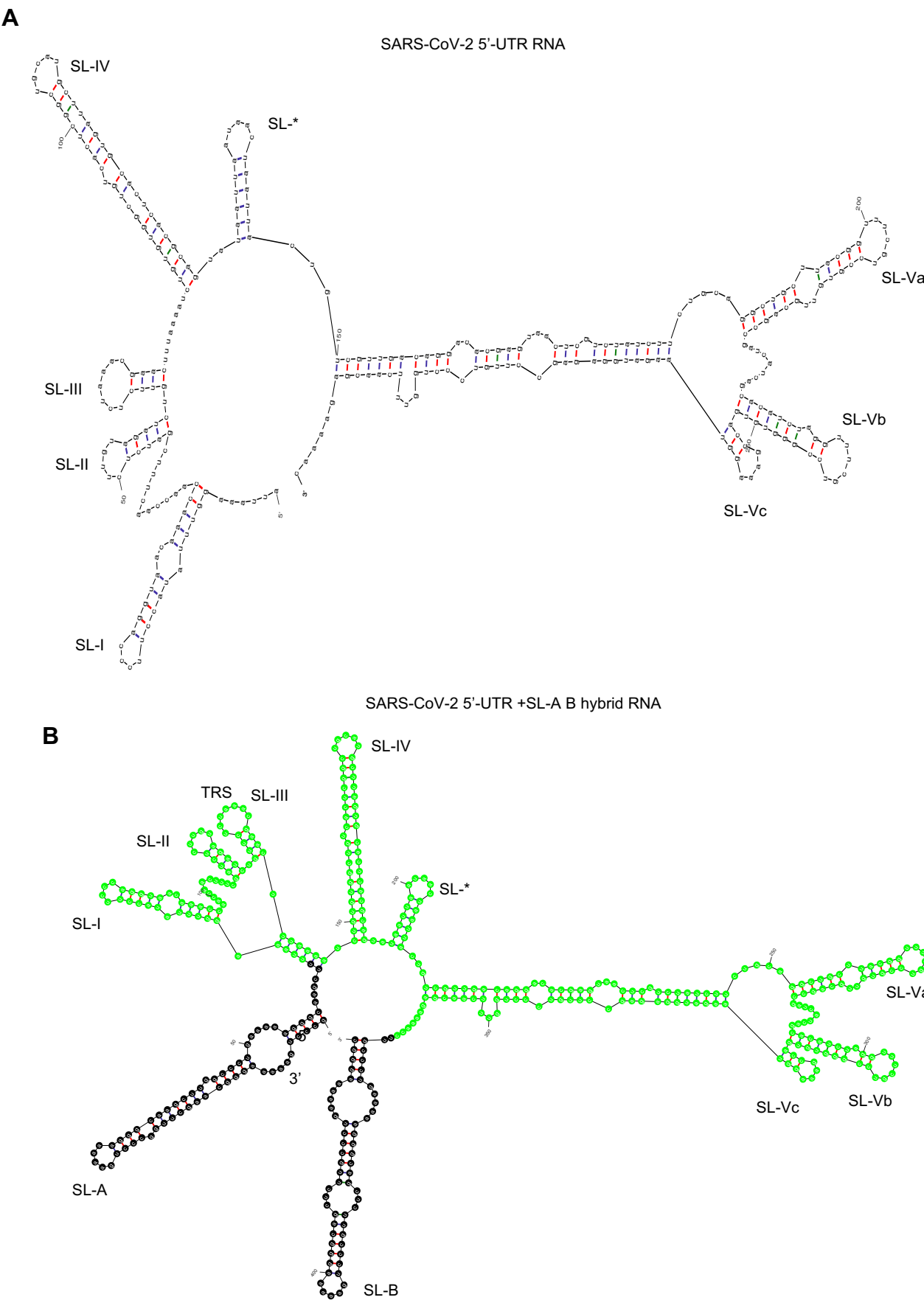

Supplement: FIG S1 [file msystems.00643-21-sf001.pdf]

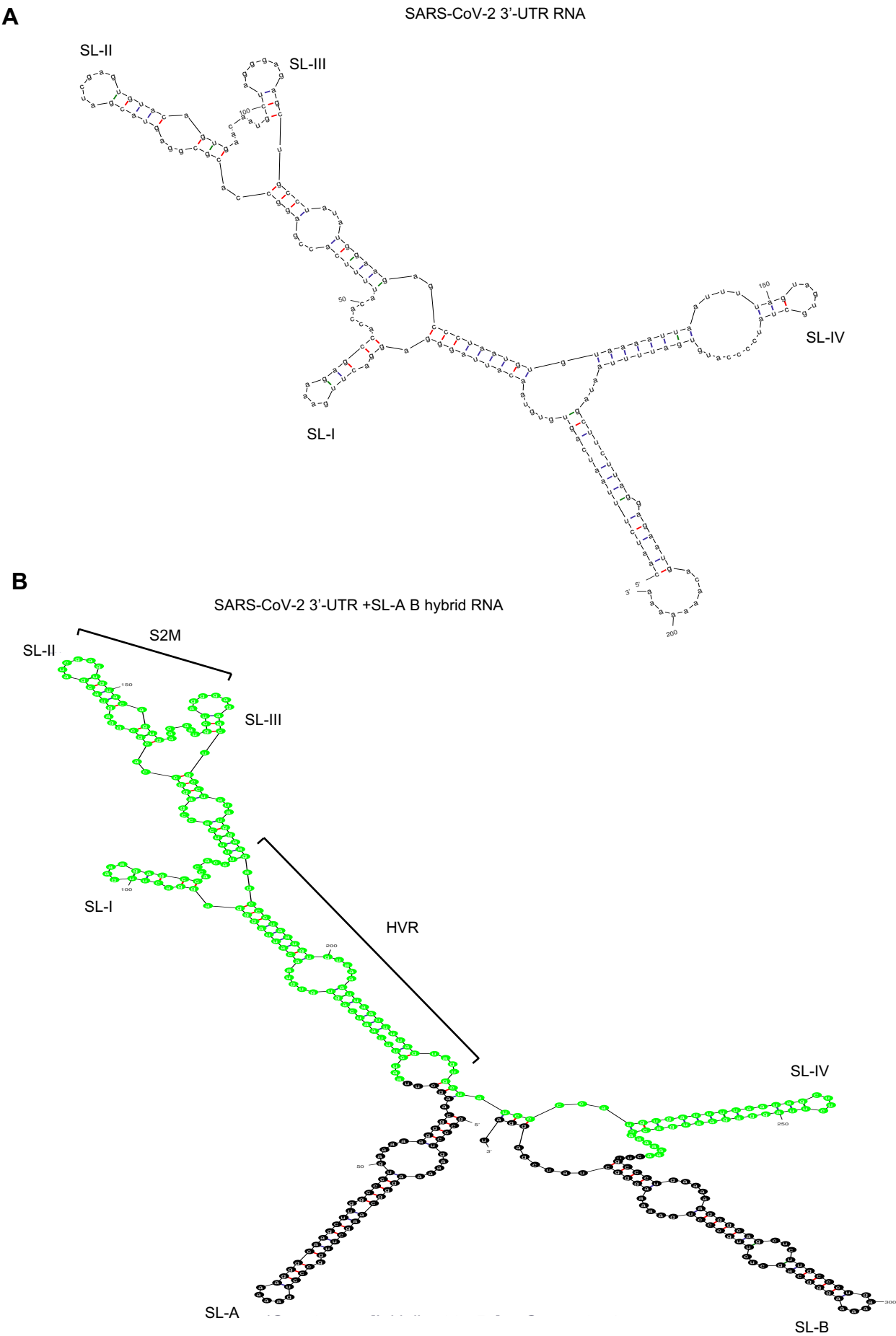

Supplement: FIG S2 [file msystems.00643-21-sf002.pdf]

Figure S6

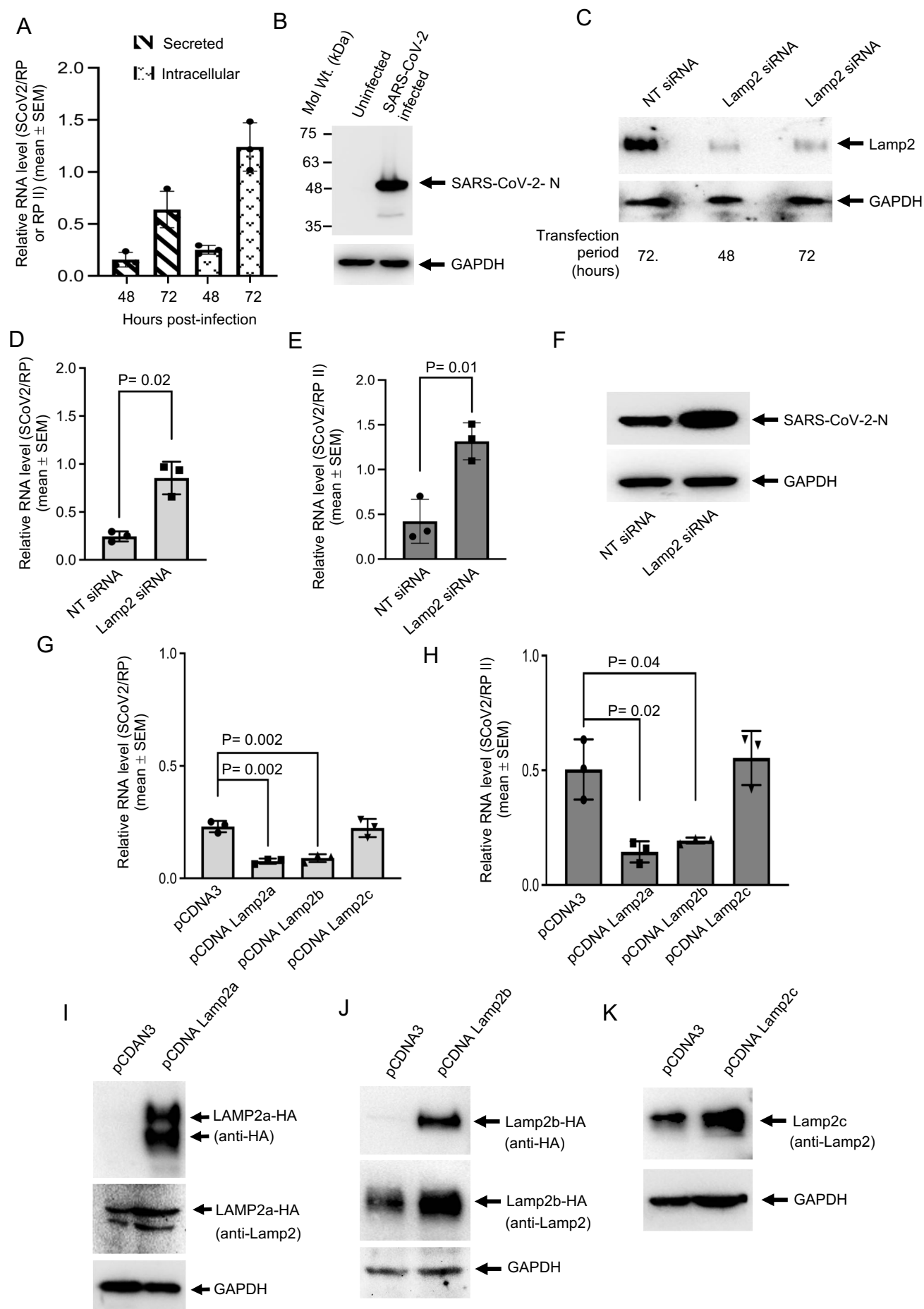

Supplement: FIG S6 [file msystems.00643-21-sf006.pdf]

Figure S7

**A**

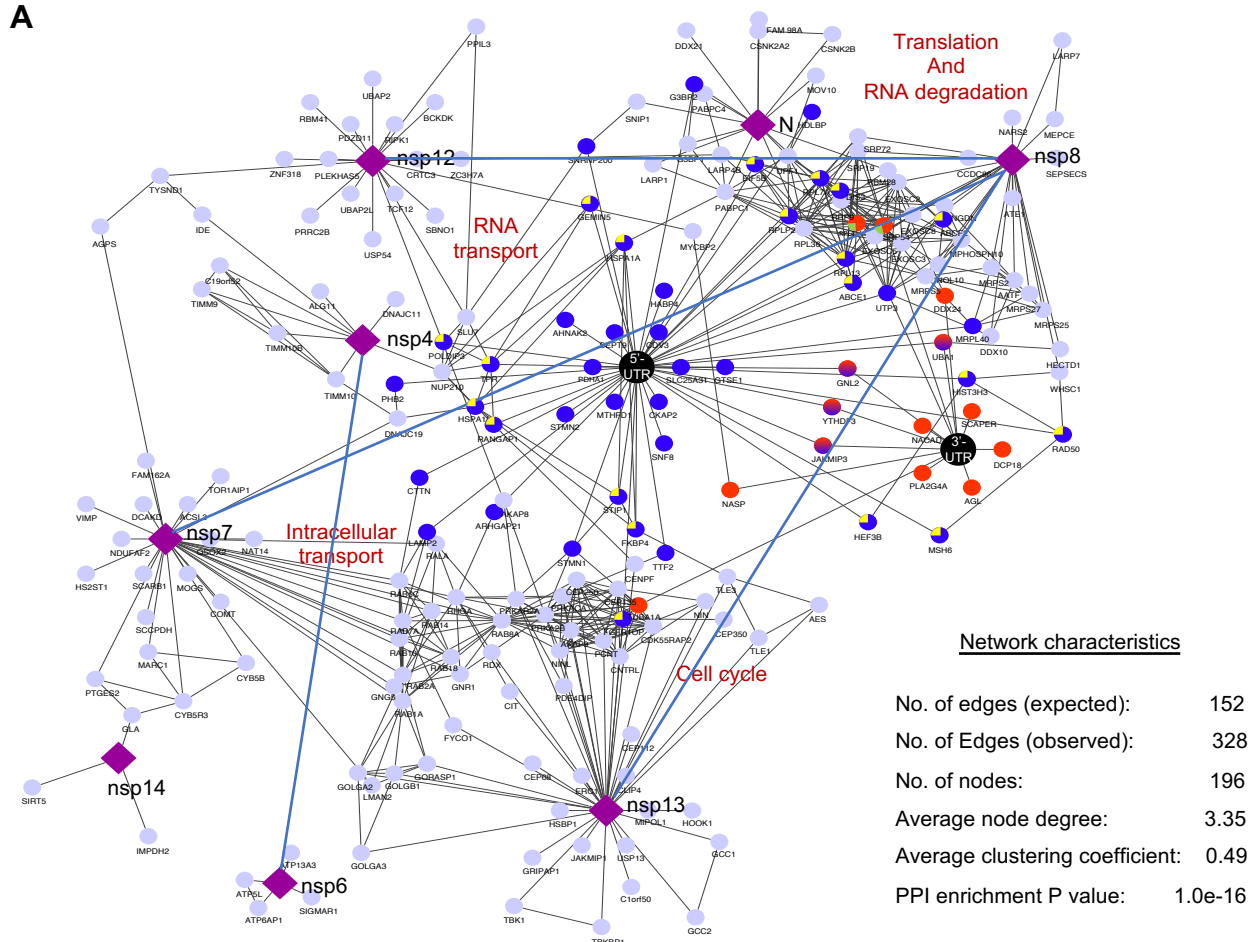

**B**

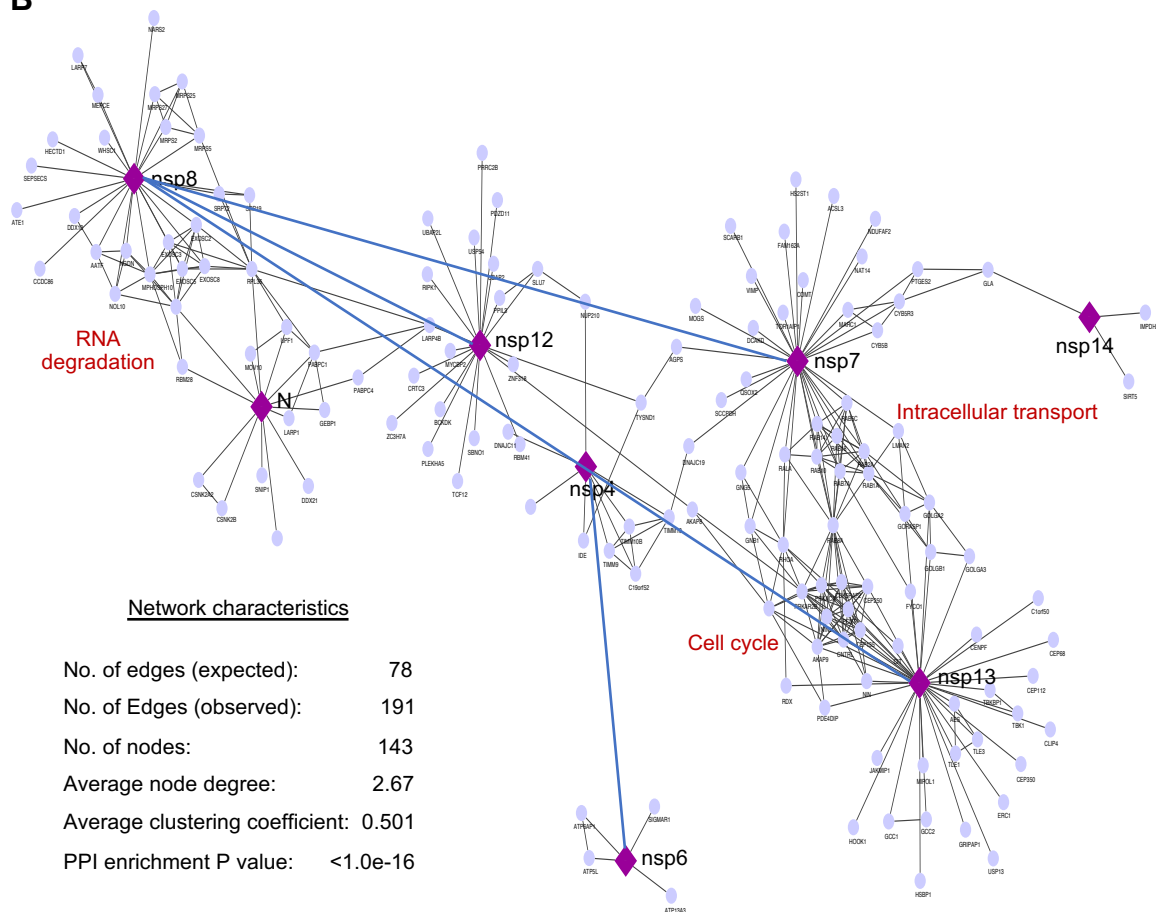

Supplement: FIG S7 [file msystems.00643-21-sf007.pdf]
