# Supplementary material for: RNA-Protein Interaction Analysis of SARS-CoV-2 5′ and 3′ Untranslated Regions Reveals a Role of Lysosome-Associated Membrane Protein-2a during Viral Infection
Source: mSystems. 2021 Jul 13;6(4):e00643-21. doi: 10.1128/mSystems.00643-21 (PMC8407388; doi:10.1128/mSystems.00643-21)
Supplement: FIG S3 [file msystems.00643-21-sf003.pdf]

Figure S3

**A**

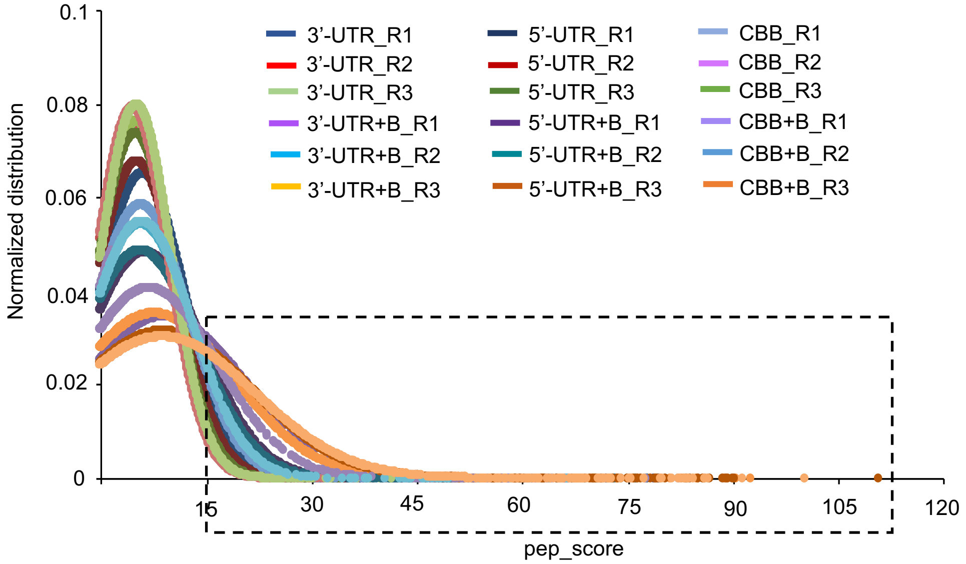


**B**

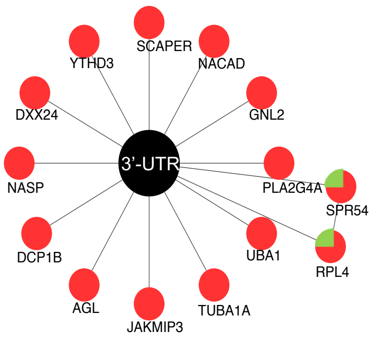


<u>Network characteristics</u>

| | |
|---|---|
| No. of edges (expected): | 1 |
| No. of Edges (observed): | 1 |
| No. of nodes: | 14 |
| Average node degree: | 0.14 |
| Average clustering coefficient: | 0.14 |
| PPI enrichment P value: | 0.437 |

**C**

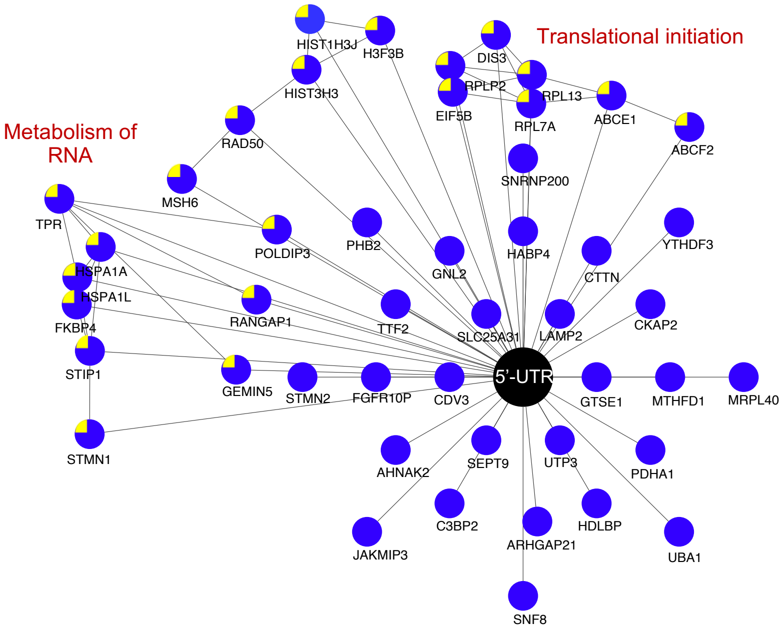


<u>Network characteristics</u>

| | |
|---|---|
| No. of edges (expected): | 10 |
| No. of Edges (observed): | 27 |
| No. of nodes: | 46 |
| Average node degree: | 1.17 |
| Average clustering coefficient: | 0.31 |
| PPI enrichment P value: | 3.64e-06 |
