# Supplementary material for: RNA-Protein Interaction Analysis of SARS-CoV-2 5′ and 3′ Untranslated Regions Reveals a Role of Lysosome-Associated Membrane Protein-2a during Viral Infection
Source: mSystems. 2021 Jul 13;6(4):e00643-21. doi: 10.1128/mSystems.00643-21 (PMC8407388; doi:10.1128/mSystems.00643-21)
Supplement: TABLE S1 [file msystems.00643-21-st001.pdf]

Table S1. List of host proteins that associate with SARS-CoV-2 5'-UTR and 3'-UTR RNA.

A. 5'-UTR RNA interacting Proteins				
**Protein name**	**prot_desc**	**prot_score**	**No. of unique peptide**	**pep_score**
AHNK2	Protein AHNAK2	45	47	48.75
KI67	Antigen KI-67	20	22	20.18
TPR	Nucleoprotein TPR	65	18	29.71
U520	U5 small nuclear ribonucleoprotein 200 kDa helicase	61	16	16.22
RHG21	Rho GTPase-activating protein 21	41	15	40.62
SETD2	Histone-lysine N-methyltransferase SETD2	24	15	24.16
VIGLN	Vigilin	46	14	40.87
AT2B1	Plasma membrane calcium-transporting ATPase 1	34	14	18.72
PDS5A	Sister chromatid cohesion protein PDS5 homolog A	16	14	15.55
RL7A	60S ribomal protein L7a	120	13	19.1
CKAP2	Cytkeleton-associated protein 2	63	13	18.93
TMF1	TATA element modulatory factor	30	13	16.3
KNTC1	Kinetochore-associated protein 1	16	13	15.81
IF2P	Eukaryotic translation initiation factor 5B	61	12	56.92
PRP8	Pre-mRNA-processing-splicing factor 8	36	12	30.62
ISK5	Serine protease inhibitor Kazal-type 5	31	12	30.53
CHD2	Chromodomain-helicase-DNA-binding protein 2	22	12	16.04
PLCB4	1-phosphatidylinitol-4,5-bisphphate phphodiesterase beta-4	15	12	15.12
TRPM6	Transient receptor potential cation channel subfamily M member 6	32	11	31.84
USP9Y	Probable ubiquitin carboxyl-terminal hydrolase FAF-Y	23	11	15.04
SMC4	Structural maintenance of chromomes protein 4	20	11	20.23
CF170	Uncharacterized protein C6orf170	17	11	19.09
HS71L	Heat shock 70 kDa protein 1-like	492	10	16.93
ZHX3	Zinc fingers and homeoboxes protein 3	33	10	15.95
GSLG1	Golgi apparatus protein 1	32	10	15.98
TRPM7	Transient receptor potential cation channel subfamily M member 7	32	10	31.84
DNMT1	DNA (cytine-5)-methyltransferase 1	30	10	18.03
THOC2	THO complex subunit 2	26	10	21.02
MAGI3	Membrane-associated guanylate kinase, WW and PDZ domain-containing protein 3	23	10	23.19
MSH6	DNA mismatch repair protein Msh6	52	9	15.59
LOXH1	Lipoxygenase homology domain-containing protein 1	31	9	30.53
TRIPB	Thyroid receptor-interacting protein 11	28	9	28.76
RFC1	Replication factor C subunit 1	22	9	22.28
DOCK2	Dedicator of cytokinesis protein 2	18	9	24.02
ALPK2	Alpha-protein kinase 2	16	9	16.15
FMN2	Formin-2	16	9	15.85
C1TC	C-1-tetrahydrofolate synthase, cytoplasmic	372	8	16.24
NOLC1	Nucleolar and coiled-body phphoprotein 1	39	8	31.63

KI13B	Kinesin-like protein KIF13B	30	8	26.58
K2022	Uncharacterized protein KIAA2022	30	8	28.99
EP400	E1A-binding protein p400	23	8	15.87
SRBP2	Sterol regulatory element-binding protein 2	23	8	23.19
TACC2	Transforming acidic coiled-coil-containing protein 2	20	8	19.31
AFF1	AF4/FMR2 family member 1	17	8	16.59
PK3CB	Phphatidylinitol-4,5-bisphphate 3-kinase catalytic subunit beta isoform	15	8	15.62
ARMC2	Armadillo repeat-containing protein 2	14	8	19.62
HSP71	Heat shock 70 kDa protein 1A/1B	278	7	21.09
RRP44	Exome complex exonuclease RRP44	180	7	15.69
ADT4	ADP/ATP translocase 4	60	7	17.73
SLIP	GTPase SLIP-GC	34	7	33.82
BLM	Bloom syndrome protein	27	7	27.42
CR021	UPF0711 protein C18orf21	25	7	27.26
AT8B1	Probable phpholipid-transporting ATPase IC	23	7	23.19
CLPB	Caseinolytic peptidase B protein homolog	22	7	17.96
CBP	CREB-binding protein	19	7	19.3
KDM5B	Lysine-specific demethylase 5B	16	7	19.43
ABCE1	ATP-binding cassette sub-family E member 1	79	6	18.26
SRC8	Src substrate cortactin	70	6	47.31
STMN2	Stathmin-2	49	6	41.43
RPAP3	RNA polymerase II-associated protein 3	34	6	34.18
LIPA3	Liprin-alpha-3	34	6	17.98
CGNL1	Cingulin-like protein 1	33	6	32.78
CJ093	TPR repeat-containing protein C10orf93	32	6	31.79
MBB1A	Myb-binding protein 1A	32	6	16.48
BRAP	BRCA1-associated protein	32	6	18.55
ABCA4	Retinal-specific ATP-binding cassette transporter	28	6	15.02
TMC1	Transmembrane channel-like protein 1	26	6	21.07
LRIQ3	Leucine-rich repeat and IQ domain-containing protein 3	23	6	23.19
GPTC1	G patch domain-containing protein 1	22	6	22.32
OLA1	Obg-like ATPase 1	20	6	20.23
MARHA	Probable E3 ubiquitin-protein ligase MARCH10	20	6	19.58
CCD66	Coiled-coil domain-containing protein 66	17	6	17.11
WDR67	WD repeat-containing protein 67	15	6	15.29
ODPA	Pyruvate dehydrogenase E1 component subunit alpha, somatic form, mitochondrial	60	5	17.08
STIP1	Stress-induced-phphoprotein 1	52	5	32.58
PDIP3	Polymerase delta-interacting protein 3	45	5	43.84
CFDP1	Craniofacial development protein 1	38	5	37.54
FMNL2	Formin-like protein 2	31	5	18.39
RGPD5	RanBP2-like and GRIP domain-containing protein 5	26	5	25.58
CHD1L	Chromodomain-helicase-DNA-binding protein 1-like	24	5	18.32
HLTF	Helicase-like transcription factor	23	5	23.19
HIRA	Protein HIRA	23	5	23.19
SHRM4	Protein Shroom4	20	5	19.82
S9A10	Sodium/hydrogen exchanger 10	19	5	18.49
GRM7	Metabotropic glutamate receptor 7	16	5	15.58

PHB2	Prohibitin-2	132	4	18.23
GTSE1	G2 and S phase-expressed protein 1	54	4	54.08
Sep-09	Septin-9	50	4	15.62
FR1OP	FGFR1 oncogene partner	49	4	35.65
STMN1	Stathmin	49	4	41.43
SAS10	Something about silencing protein 10	41	4	37.68
FKBP4	Peptidyl-prolyl cis-trans isomerase FKBP4	40	4	18.94
CA141	Uncharacterized protein C1orf141	35	4	35.33
PROS	Vitamin K-dependent protein S	35	4	20.23
MBD4	Methyl-CpG-binding domain protein 4	33	4	33.1
QRIC2	Glutamine-rich protein 2	31	4	30.53
GLYL2	Glycine N-acyltransferase-like protein 2	31	4	30.59
MBD5	Methyl-CpG-binding domain protein 5	30	4	17.06
PAB4L	Polyadenylate-binding protein 4-like	27	4	16.9
NU133	Nuclear pore complex protein Nup133	24	4	24.44
DDX59	Probable ATP-dependent RNA helicase DDX59	23	4	23.19
PAPP2	Pappalysin-2	23	4	23.19
K0408	Uncharacterized protein KIAA0408	23	4	23.24
SHAN3	SH3 and multiple ankyrin repeat domains protein 3	23	4	15.27
CDK14	Cell division protein kinase 14	22	4	20.65
RAIN	Ras-interacting protein 1	21	4	15.66
DBF4A	Protein DBF4 homolog A	18	4	21.02
MIPT3	TRAF3-interacting protein 1	18	4	18.18
RGPD2	RanBP2-like and GRIP domain-containing protein 2	18	4	16.36
DTX3L	E3 ubiquitin-protein ligase DTX3L	18	4	17.73
PSD12	26S proteasome non-ATPase regulatory subunit 12	18	4	17.92
RBGPR	Rab3 GTPase-activating protein non-catalytic subunit	16	4	15.18
CQ057	EF-hand domain-containing protein C17orf57	16	4	16.43
ZNF92	Zinc finger protein 92	16	4	16
DOCK3	Dedicator of cytokinesis protein 3	16	4	16.15
ZN273	Zinc finger protein 273	16	4	16
ZN506	Zinc finger protein 506	16	4	16
ATS9	A disintegrin and metalloproteinase with thrombpondin motifs 9	16	4	16.41
RGS3	Regulator of G-protein signaling 3	16	4	16.42
TM63B	Transmembrane protein 63B	16	4	16.03
H31T	Histone H3.1t	212	3	16.3
H31	Histone H3.1	212	3	16.3
H33	Histone H3.3	212	3	16.3
SNF8	Vacuolar-sorting protein SNF8	104	3	70.18
GEMI5	Gem-associated protein 5	55	3	54.58
RM40	39S ribomal protein L40, mitochondrial	49	3	16.9
RAGP1	Ran GTPase-activating protein 1	49	3	15.03
TTF2	Transcription termination factor 2	48	3	46.28
CDV3	Protein CDV3 homolog	41	3	41.3
NOP16	Nucleolar protein 16	35	3	33.72
MS3L2	Putative male-specific lethal-3 protein-like 2	31	3	30.59
DPP10	Inactive dipeptidyl peptidase 10	29	3	21.86
ZN257	Zinc finger protein 257	28	3	28.08

K0819	Uncharacterized protein KIAA0819	27	3	16.21
ARP19	cAMP-regulated phphoprotein 19	26	3	22.93
RGPD3	RanBP2-like and GRIP domain-containing protein 3	26	3	25.58
CCNT2	Cyclin-T2	24	3	24
GRP3	Ras guanyl-releasing protein 3	24	3	15.84
KLH35	Kelch-like protein 35	22	3	18.78
PRAM7	PRAME family member 7	22	3	15.16
RL3	60S ribomal protein L3	22	3	28.64
ZN711	Zinc finger protein 711	21	3	18.42
TGS1	Trimethylguanine synthase	20	3	20.57
STRBP	Spermatid perinuclear RNA-binding protein	20	3	16.58
CJ071	Uncharacterized protein C10orf71	18	3	18.34
CJ111	Uncharacterized protein C10orf111	17	3	17.08
RASL3	RAS protein activator like-3	17	3	17.36
CCR7	C-C chemokine receptor type 7	16	3	20.02
ZNF93	Zinc finger protein 93	16	3	16
TM38B	Trimeric intracellular cation channel type B	15	3	17.45
LAMP2	Lysome-associated membrane glycoprotein 2	119	2	16.22
RLA2	60S acidic ribomal protein P2	110	2	18.72
G3BP2	Ras GTPase-activating protein-binding protein 2	98	2	23.99
HS902	Putative heat shock protein HSP 90-alpha A2	77	2	17.6
ABCF2	ATP-binding cassette sub-family F member 2	70	2	57.85
HABP4	Intracellular hyaluronan-binding protein 4	41	2	39.34
PKHF2	Pleckstrin homology domain-containing family F member 2	38	2	16.48
H6ST3	Heparan-sulfate 6-O-sulfotransferase 3	37	2	37.15
COASY	Bifunctional coenzyme A synthase	36	2	17.5
PDLI1	PDZ and LIM domain protein 1	36	2	35.82
FA83H	Protein FAM83H	32	2	32.25
CRBL2	cAMP-responsive element-binding protein-like 2	31	2	30.53
CF201	Uncharacterized protein C6orf201	30	2	30.46
2AAB	Serine/threonine-protein phphatase 2A 65 kDa regulatory subunit A beta isoform	27	2	26.86
RGPD4	RanBP2-like and GRIP domain-containing protein 4	26	2	25.58
TAGAP	T-cell activation Rho GTPase-activating protein	26	2	26.23
LIN41	Tripartite motif-containing protein 71	25	2	24.65
NRIP2	Nuclear receptor-interacting protein 2	25	2	25.11
MFAP3	Microfibril-associated glycoprotein 3	24	2	24.03
SMU1	WD40 repeat-containing protein SMU1	23	2	23.26
ZN347	Zinc finger protein 347	23	2	23.19
T132B	Transmembrane protein 132B	23	2	23.19
BOREA	Borealin	22	2	22.47
TBR1	T-box brain protein 1	22	2	16.14
SLIK2	SLIT and NTRK-like protein 2	21	2	24.33
PRAP1	Proline-rich acidic protein 1	20	2	19.15
VPS35	Vacuolar protein sorting-associated protein 35	19	2	15.04
SPXN3	Sperm protein associated with the nucleus on the X chromome N3	19	2	18.88
STRN	Striatin	19	2	18.76
MAST1	Microtubule-associated serine/threonine-protein kinase 1	18	2	17.62

ZN397	Zinc finger protein 397	18	2	18.2
MYOZ3	Myozenin-3	17	2	18.95
PANX2	Pannexin-2	17	2	16.64
BRAF	Serine/threonine-protein kinase B-raf	16	2	15.06
ZN680	Zinc finger protein 680	16	2	16
PCDBG	Protocadherin beta-16	15	2	23.49
TBA1C	Tubulin alpha-1C chain	557	1	38.23
H4	Histone H4	412	1	17.21
H3L	Histone H3-like	169	1	17.89
HNRDL	Heterogeneous nuclear ribonucleoprotein D-like	165	1	17.94
TAAR2	Trace amine-associated receptor 2	56	1	20.08
SSR2	Somattatin receptor type 2	39	1	38.57
CCDC6	Coiled-coil domain-containing protein 6	35	1	16.07
NUPL2	Nucleoporin-like protein 2	35	1	35.65
BODG	Gamma-butyrobetaine dioxygenase	31	1	30.53
HMN2L	Putative non-histone chromomal protein HMG-17-like	31	1	30.53
TXND9	Thioredoxin domain-containing protein 9	28	1	27.66
ILK	Integrin-linked protein kinase	26	1	25.52
MFA3L	Microfibrillar-associated protein 3-like	24	1	24.03
O10AG	Olfactory receptor 10AG1	24	1	18.37
ERG7	Lanterol synthase	24	1	23.61
ANR19	Ankyrin repeat domain-containing protein 19	23	1	23.19
F155B	Transmembrane protein FAM155B	23	1	23.19
ITIH5	Inter-alpha-trypsin inhibitor heavy chain H5	23	1	23.26
MERTK	Tyrine-protein kinase Mer	23	1	16.87
TIG3	Retinoic acid receptor responder protein 3	19	1	18.52
ARHG2	Rho guanine nucleotide exchange factor 2	18	1	18.17
CI068	Uncharacterized protein C9orf68	18	1	17.51
KLH20	Kelch-like protein 20	16	1	16.23
SC5A5	Sodium/iodide cotransporter	16	1	20.02
P2RX3	P2X purinoceptor 3	15	1	15.28
I17RE	Interleukin-17 receptor E	14	1	15.78
MIRH1	Putative microRNA 17 ht gene protein	14	1	17.57

B. 3'-UTR RNA interacting Proteins

MACF4	Microtubule-actin crs-linking factor 1, isoform 4	34	70	18.09
MACF1	Microtubule-actin crs-linking factor 1, isoforms 1/2/3/5	34	65	18.09
BPAEA	Bullous pemphigoid antigen 1, isoforms 6/9/10	29	47	18.63
FAT4	Protocadherin Fat 4	38	31	15.98
ANR12	Ankyrin repeat domain-containing protein 12	20	28	24.93
C8AP2	CASP8-associated protein 2	36	21	22.65
TEX15	Testis-expressed sequence 15 protein	31	21	19.44
PCLO	Protein piccolo	27	20	27.13
PIBF1	Progesterone-induced-blocking factor 1	23	18	23.1
DYH2	Dynein heavy chain 2, axonemal	33	17	21.78
DDX24	ATP-dependent RNA helicase DDX24	40	15	19
SNTB2	Beta-2-syntrophin	27	15	16.1
CO6A5	Collagen alpha-5(VI) chain	26	15	17.6
CX022	Uncharacterized protein CXorf22	26	15	15.3

NACAD	NAC-alpha domain-containing protein 1	44	14	16.16
AT10D	Probable phpholipid-transporting ATPase VD	39	14	36.84
VPS39	Vam6/Vps39-like protein	35	13	31.1
DPOLQ	DNA polymerase theta	18	13	21.77
VPS52	Vacuolar protein sorting-associated protein 52 homolog	16	12	15.99
CG025	UPF0415 protein C7orf25	29	11	29.07
SMRCD	SWI/SNF-related matrix-associated actin-dependent regulator of chromatin subfamily A containing DEAD/H box 1	17	11	17.47
CE110	Centromal protein of 110 kDa	18	10	15.77
PHF2	PHD finger protein 2	24	9	24.46
MAP1B	Microtubule-associated protein 1B	22	9	15.23
UIF	UAP56-interacting factor	18	9	18.42
COG4	Conserved oligomeric Golgi complex subunit 4	17	9	17.25
AT10B	Probable phpholipid-transporting ATPase VB	39	8	36.84
K1143	Uncharacterized protein KIAA1143	29	8	29.07
LIMA1	LIM domain and actin-binding protein 1	26	8	26.26
RABX5	Rab5 GDP/GTP exchange factor	24	8	18.52
K0090	Uncharacterized protein KIAA0090	22	8	15.81
ASB3	Ankyrin repeat and SOCS box protein 3	23	7	15.41
ZN592	Zinc finger protein 592	23	7	22.97
PIM1	Proto-oncogene serine/threonine-protein kinase pim-1	18	7	17.59
RBM43	RNA-binding protein 43	18	7	17.53
CCD25	Coiled-coil domain-containing protein 25	16	7	15.78
TICRR	TopBP1-interacting checkpoint and replication regulator	29	6	18.37
GBA2	Non-lysomal glucylceramidase	27	6	27.13
KS6B2	Ribomal protein S6 kinase beta-2	24	6	24.16
FGD6	FYVE, RhoGEF and PH domain-containing protein 6	15	6	15.08
TBA1A	Tubulin alpha-1A chain	536	5	24.25
KCNKA	Potassium channel subfamily K member 10	27	5	17.51
DBX2	Homeobox protein DBX2	26	5	26.14
ASPN	Asporin	24	5	24.04
TEX10	Testis-expressed sequence 10 protein	22	5	21.87
AL1A3	Aldehyde dehydrogenase family 1 member A3	21	5	20.64
TEX2	Testis-expressed sequence 2 protein	19	5	18.79
PDZD7	PDZ domain-containing protein 7	13	5	16.7
PA24A	Cytolic phpholipase A2	53	4	17.26
DCP1B	mRNA-decapping enzyme 1B	51	4	35.12
TLR3	Toll-like receptor 3	31	4	31.39
TTMA	Transmembrane protein TTMA	29	4	29.32
CATA	Catalase	26	4	25.63
SCMC1	Calcium-binding mitochondrial carrier protein SCaMC-1	26	4	16.73
CCNK	Cyclin-K	22	4	22.41
TNIK	TRAF2 and NCK-interacting protein kinase	22	4	22.13
CTNA3	Catenin alpha-3	18	4	17.77
PIGG	GPI ethanolamine phphate transferase 2	18	4	17.85
SPD2A	SH3 and PX domain-containing protein 2A	18	4	18.25
ZMYM3	Zinc finger MYM-type protein 3	18	4	18.18
RHG23	Rho GTPase-activating protein 23	16	4	17.53
NASP	Nuclear autoantigenic sperm protein	218	3	15.02

VGLU2	Vesicular glutamate transporter 2	29	3	28.55
CADH9	Cadherin-9	28	3	28
SH3B4	SH3 domain-binding protein 4	28	3	28.14
WDR90	WD repeat-containing protein 90	26	3	15.32
PECA1	Platelet endothelial cell adhesion molecule	24	3	23.72
SCND3	SCAN domain-containing protein 3	20	3	20.01
M4K4	Mitogen-activated protein kinase kinase kinase kinase 4	18	3	16.5
MAFIP	MaFF-interacting protein	16	3	16.23
RERGL	Ras-related and estrogen-regulated growth inhibitor-like protein	15	3	15.14
GRAM4	GRAM domain-containing protein 4	29	2	28.55
BTBD7	BTB/POZ domain-containing protein 7	24	2	24.16
EHMT1	Histone-lysine N-methyltransferase, H3 lysine-9 specific 5	24	2	24.16
RS17	40S ribomal protein S17	18	2	17.29
HAND2	Heart- and neural crest derivatives-expressed protein 2	102	1	15.36
RM11	39S ribomal protein L11, mitochondrial	36	1	35.94
ENPP4	Ectonucleotide pyrophphatase/phphodiesterase family member 4	18	1	18.18
BCAM	Basal cell adhesion molecule	16	1	16.41
DEPD6	DEP domain-containing mTOR-interacting protein	16	1	15.96
KDM7A	Histone lysine demethylase JHDM1D	24	10	24.46
