# Supplementary material for: RNA-Protein Interaction Analysis of SARS-CoV-2 5′ and 3′ Untranslated Regions Reveals a Role of Lysosome-Associated Membrane Protein-2a during Viral Infection
Source: mSystems. 2021 Jul 13;6(4):e00643-21. doi: 10.1128/mSystems.00643-21 (PMC8407388; doi:10.1128/mSystems.00643-21)
Supplement: TABLE S2 [file msystems.00643-21-st002.pdf]

Table S2. Host proteins that interact with the 3'-end of HEV genome, identified by RaPID assay.

Gene ID	Protein name	Prot_score	No. of biotinylated peptides	No. of unique peptides
FUBP3	Far upstream element-binding protein 3	1010	5	4
GAPDH	Glyceraldehyde-3-phphate dehydrogenase	665	3	3
TNPO1	Transportin-1	649	15	7
TUBA3C	Tubulin alpha-3C/D chain	420	4	3
RPSA	40S ribomal protein SA	386	6	4
KHSRP	Far upstream element-binding protein 2	354	8	7
CTPS2	CTP synthase 2	273	13	10
RUVBL2	RuvB-like 2	270	7	7
CCT8	T-complex protein 1 subunit theta	264	11	9
KPNA5	Importin subunit alpha-6	231	10	9
HIST1H3A	Histone H3.1	210	4	4
TNPO2	Transportin-2	210	16	8
LDHC	L-lactate dehydrogenase C chain	187	8	6
HNRPDL	Heterogeneous nuclear ribonucleoprotein D-like	155	9	7
RBM27	RNA-binding protein 27	151	36	24
PIN4	Peptidyl-prolyl cis-trans isomerase NIMA-interacting 4	145	7	7
RBM39	RNA-binding protein 39	143	5	5
BANF1	Barrier-to-autointegration factor	136	2	2
KPNA4	Importin subunit alpha-4	133	4	3
HPRT1	Hypoxanthine-guanine phphoribyltransferase	131	6	5
SOD1	Superoxide dismutase [Cu-Zn]	108	5	4
C6orf132	Uncharacterized protein C6orf132	106	18	10
SLC25A11	Mitochondrial 2-oxoglutarate/malate carrier protein	102	3	3
NIT2	Omega-amidase NIT2	101	10	4
AKR1C4	Aldo-keto reductase family 1 member C4	99	11	7
SNX2	Sorting nexin-2	97	11	9
TJP1	Tight junction protein ZO-1	93	10	9
CAPZB	F-actin-capping protein subunit beta	88	4	4
HSPH1	Heat shock protein 105 kDa	87	7	6
RELA	Transcription factor p65	87	4	4
HMGB3L1	High mobility group protein B3-like-1	84	12	8
AKR1A1	Alcohol dehydrogenase [NADP+]	75	5	5
AKR1B1	Alde reductase	75	10	8
AK1	Adenylate kinase isoenzyme 1	74	5	5
MCM4	DNA replication licensing factor MCM4	69	9	7
TPM1	Tropomyin alpha-1 chain	66	15	7
TPM2	Tropomyin beta chain	66	14	5
ZFAND1	AN1-type zinc finger protein 1	66	15	8
FMR1	Fragile X mental retardation 1 protein	61	7	6
PSMD8	26S proteasome non-ATPase regulatory subunit 8	59	4	3
KIAA1143	Uncharacterized protein KIAA1143	53	4	3
DNAJC7	DnaJ homolog subfamily C member 7	52	4	2
DCAKD	Dephpho-CoA kinase domain-containing protein	48	6	3
ARHGDIA	Rho GDP-dissociation inhibitor 1	48	6	5
TMED9	Transmembrane emp24 domain-containing protein 9	48	5	2
HARS2	Probable histidyl-tRNA synthetase, mitochondrial	47	3	2
CFL2	Cofilin-2	46	3	3
SCP2	Non-specific lipid-transfer protein	46	21	13
PSMD7	26S proteasome non-ATPase regulatory subunit 7	45	10	6
TNKS2	Tankyrase-2	45	23	20
PGM2	Phphoglucomutase-2	42	10	2
SEPT11	Septin-11	41	16	10
SEPT14	Septin-14	41	14	9
SEPT7	Septin-7	41	13	12
KRT32	Keratin, type I cuticular Ha2	40	2	2
