# Supplementary material for: RNA-Protein Interaction Analysis of SARS-CoV-2 5′ and 3′ Untranslated Regions Reveals a Role of Lysosome-Associated Membrane Protein-2a during Viral Infection
Source: mSystems. 2021 Jul 13;6(4):e00643-21. doi: 10.1128/mSystems.00643-21 (PMC8407388; doi:10.1128/mSystems.00643-21)
Supplement: TABLE S3 [file msystems.00643-21-st003.pdf]

Table S3. Comparison of expression of prey proteins identified in RaPID assay in human Lungs and Intestine.

Gene name	Lungs	Intestine
ABCE1	35.3	26.2
ABCF2	21.6	13.8
AGL	11.6	10.8
AHNAK2	6.1	2.5
ARHGAP21	45	26.3
CDV3	165.6	91.3
CKAP2	10.3	14.9
CTTN	91.5	85.8
DCP1B	7.7	4.7
DDX24	9.6	2
DIS3	22.7	15.3
EIF5B	44.7	32.5
FGFR1OP	14.7	24.3
FKBP4	31.8	39.3
G3BP2	101.1	104.7
GEMIN5	8.6	4.4
GNL2	41.1	27.4
GTSE1	0.9	4.4
H3F3A	841	1020.8
HABP4	13.5	5.4
HDLBP	222.7	202.6
HIST1H3A	0.9	0.4
HIST3H3	0	0
HSPA1A	56.8	12.1
HSPA1L	0.5	0.3
JAKMIP3	0.3	0.1
LAMP2	168.4	108.2
MRPL40	14.4	12
MSH6	26.9	15.8
MTHFD1	34.9	35
NACAD	2.8	1.9
NASP	70.4	61.4
PDHA1	63.6	126.7
PHB2	133.3	179.2
PLA2G4A	11.7	12.7
POLDIP3	65	37
RAD50	5.6	6.9
RANGAP1	34.7	32.5
RPL13	1275.1	1479.3

RPL4	1316.3	752.2
RPL7A	854.7	764.3
RPLP2	600.3	767.1
SCAPER	12.6	9.7
SEPT9.	135.9	182.5
SLC25A31	0	0
SNF8	36.5	36.6
SNRNP200	57.1	45.3
SRP54	54	46.4
STIP1	91.7	66.7
STMN1	119.4	137.8
STMN2	0.5	27.9
TPR	35.5	24.5
TTF2	5.2	6.2
TUBA1A	422.7	110.6
UBA1	42.07	37.9
UTP3	35.6	25
YTHDF3	38.8	33.7
Values represent pTPM scores of RNA seq data, collected from the human protein atlas and the expression atlas.		
