# Supplementary material for: RNA-Protein Interaction Analysis of SARS-CoV-2 5′ and 3′ Untranslated Regions Reveals a Role of Lysosome-Associated Membrane Protein-2a during Viral Infection
Source: mSystems. 2021 Jul 13;6(4):e00643-21. doi: 10.1128/mSystems.00643-21 (PMC8407388; doi:10.1128/mSystems.00643-21)
Supplement: FIG S4 [file msystems.00643-21-sf004.pdf]

Figure S4

Entrez Gene Id	Gene Symbol	GO_INTRACELLULAR_TRANSPORT	GO_INTRACELLULAR_PROTEIN_TRANSPORT	GO_TRANSLATIONAL_INITIATION	GO_AMIDE_BIOSYNTHETIC_PROCESS	GO_PROTEIN_CONTAINING_COMPLEX_SUBUNIT_ORGANIZATION	GO_MRNA_METABOLIC_PROCESS	GO_PEPTIDE_BIOSYNTHETIC_PROCESS	GO_CELLULAR_AMIDE_METABOLIC_PROCESS	GO_CELLULAR_MACROMOLECULE_LOCALIZATION	GO_PEPTIDE_METABOLIC_PROCESS	Entrez	Ensembl	Gene Description
7175	TPR	■	■	■	■	■		■	■	■	■			translocated promoter region, nuclear basket protein [Source:HGNC Symbol;Acc:HGNC:12017]
6059	ABCE1	■	■	■	■	■		■	■	■	■			ATP binding cassette subfamily E member 1 [Source:HGNC Symbol;Acc:HGNC:69]
6130	RPL7A	■	■	■	■		■	■	■	■	■			ribosomal protein L7a [Source:HGNC Symbol;Acc:HGNC:10364]
6181	RPLP2	■	■	■	■		■	■	■	■	■			ribosomal protein lateral stalk subunit P2 [Source:HGNC Symbol;Acc:HGNC:10377]
6137	RPL13	■	■	■	■		■	■	■	■	■			ribosomal protein L13 [Source:HGNC Symbol;Acc:HGNC:10303]
6124	RPL4	■	■	■	■		■	■	■	■	■			ribosomal protein L4 [Source:HGNC Symbol;Acc:HGNC:10353]
84271	POLDIP3	■	■		■		■	■	■	■	■			DNA polymerase delta interacting protein 3 [Source:HGNC Symbol;Acc:HGNC:23782]
6729	SRP54	■	■			■				■				signal recognition particle 54 [Source:HGNC Symbol;Acc:HGNC:11301]
3920	LAMP2	■	■			■				■				lysosomal associated membrane protein 2 [Source:HGNC Symbol;Acc:HGNC:6501]
2017	CTTN	■	■			■				■				cortactin [Source:HGNC Symbol;Acc:HGNC:3338]
11331	PHB2	■	■							■				prohibitin 2 [Source:HGNC Symbol;Acc:HGNC:30306]
11267	SNF8	■	■							■				SNF8 subunit of ESCRT-II [Source:HGNC Symbol;Acc:HGNC:17028]
3305	HSPA1L	■	■							■				heat shock protein family A (Hsp70) member 1 like [Source:HGNC Symbol;Acc:HGNC:5234]
51512	GTSE1	■	■							■				G2 and S-phase expressed 1 [Source:HGNC Symbol;Acc:HGNC:13698]
5905	RANGAP1	■	■							■				Ran GTPase activating protein 1 [Source:HGNC Symbol;Acc:HGNC:9854]
23148	NACAD	■	■							■				NAC alpha domain containing [Source:HGNC Symbol;Acc:HGNC:22196]
25929	GEMIN5	■			■	■	■	■	■		■			gem nuclear organelle associated protein 5 [Source:HGNC Symbol;Acc:HGNC:20043]
7846	TUBA1A	■												tubulin alpha 1a [Source:HGNC Symbol;Acc:HGNC:20766]
57584	ARHGAP21	■												Rho GTPase activating protein 21 [Source:HGNC Symbol;Acc:HGNC:23725]
253943	YTHDF3			■	■		■	■	■		■			YTH N6-methyladenosine RNA binding protein 3 [Source:HGNC Symbol;Acc:HGNC:26465]
22927	HABP4			■	■		■	■	■		■			hyaluronan binding protein 4 [Source:HGNC Symbol;Acc:HGNC:17062]
9669	EIF5B			■	■			■	■		■			eukaryotic translation initiation factor 5B [Source:HGNC Symbol;Acc:HGNC:30793]
64976	MRPL40				■	■		■	■		■			mitochondrial ribosomal protein L40 [Source:HGNC Symbol;Acc:HGNC:14491]
5160	PDHA1				■				■					pyruvate dehydrogenase E1 subunit alpha 1 [Source:HGNC Symbol;Acc:HGNC:8806]
4522	MTHFD1				■				■					methylenetetrahydrofolate dehydrogenase, cyclohydrolase and formyltetrahydrofolate synthetase 1 [Source:HGNC Symbol;Acc:HGNC:7432]
3303	HSPA1A					■	■							heat shock protein family A (Hsp70) member 1A [Source:HGNC Symbol;Acc:HGNC:5232]
23020	SNRNP200					■	■							small nuclear ribonucleoprotein U5 subunit 200 [Source:HGNC Symbol;Acc:HGNC:30859]
9908	G3BP2					■								G3BP stress granule assembly factor 2 [Source:HGNC Symbol;Acc:HGNC:30291]
11075	STMN2					■								stathmin 2 [Source:HGNC Symbol;Acc:HGNC:10577]
3925	STMN1					■								stathmin 1 [Source:HGNC Symbol;Acc:HGNC:6510]
2288	FKBP4					■								FKBP prolyl isomerase 4 [Source:HGNC Symbol;Acc:HGNC:3720]
3020	H3-3A					■								H3.3 histone A [Source:HGNC Symbol;Acc:HGNC:4764]
26586	CKAP2					■								cytoskeleton associated protein 2 [Source:HGNC Symbol;Acc:HGNC:1990]
4678	NASP					■								nuclear autoantigenic sperm protein [Source:HGNC Symbol;Acc:HGNC:7644]
8350	H3C1					■								H3 clustered histone 1 [Source:HGNC Symbol;Acc:HGNC:4766]
8290	H3-4					■								H3.4 histone [Source:HGNC Symbol;Acc:HGNC:4778]
196513	DCP1B						■							decapping mRNA 1B [Source:HGNC Symbol;Acc:HGNC:24451]
22894	DIS3						■							DIS3 homolog, exosome endoribonuclease and 3'-5' exoribonuclease [Source:HGNC Symbol;Acc:HGNC:20604]
8458	TTF2						■							transcription termination factor 2 [Source:HGNC Symbol;Acc:HGNC:12398]
10801	SEPTIN9									■				septin 9 [Source:HGNC Symbol;Acc:HGNC:7323]
10111	RAD50													RAD50 double strand break repair protein [Source:HGNC Symbol;Acc:HGNC:9816]
2956	MSH6													mutS homolog 6 [Source:HGNC Symbol;Acc:HGNC:7329]
5321	PLA2G4A													phospholipase A2 group IVA [Source:HGNC Symbol;Acc:HGNC:9035]
11116	CEP43													centrosomal protein 43 [Source:HGNC Symbol;Acc:HGNC:17012]
7317	UBA1													ubiquitin like modifier activating enzyme 1 [Source:HGNC Symbol;Acc:HGNC:12469]
178	AGL													amylo-alpha-1, 6-glucosidase, 4-alpha-glucanotransferase [Source:HGNC Symbol;Acc:HGNC:321]
57050	UTP3													UTP3 small subunit processome component [Source:HGNC Symbol;Acc:HGNC:24477]
29889	GNL2													G protein nucleolar 2 [Source:HGNC Symbol;Acc:HGNC:29925]
113146	AHNAK2													AHNAK nucleoprotein 2 [Source:HGNC Symbol;Acc:HGNC:20125]
83447	SLC25A31													solute carrier family 25 member 31 [Source:HGNC Symbol;Acc:HGNC:25319]
10963	STIP1													stress induced phosphoprotein 1 [Source:HGNC Symbol;Acc:HGNC:11387]
3069	HDLBP													high density lipoprotein binding protein [Source:HGNC Symbol;Acc:HGNC:4857]
10061	ABCF2													ATP binding cassette subfamily F member 2 [Source:HGNC Symbol;Acc:HGNC:71]
282973	JAKMIP3													Janus kinase and microtubule interacting protein 3 [Source:HGNC Symbol;Acc:HGNC:23523]
49855	SCAPER													S-phase cyclin A associated protein in the ER [Source:HGNC Symbol;Acc:HGNC:13081]
55573	CDV3													CDV3 homolog [Source:HGNC Symbol;Acc:HGNC:26928]
57062	DDX24													DEAD-box helicase 24 [Source:HGNC Symbol;Acc:HGNC:13266]
