# Supplementary material for: RNA-Protein Interaction Analysis of SARS-CoV-2 5′ and 3′ Untranslated Regions Reveals a Role of Lysosome-Associated Membrane Protein-2a during Viral Infection
Source: mSystems. 2021 Jul 13;6(4):e00643-21. doi: 10.1128/mSystems.00643-21 (PMC8407388; doi:10.1128/mSystems.00643-21)
Supplement: FIG S5 [file msystems.00643-21-sf005.pdf]

Figure S5

Entrez Gene Id	Gene Symbol	REACTOME_CELLULAR_RESPONSES_TO_EXTERNAL_STIMULI	REACTOME_METABOLISM_OF_RNA	REACTOME_HSP90_CHAPERONE_CYCLE_FOR_STEROID_HORMONE_RECEPTORS_SHR_	REACTOME_INFLUENZA_INFECTION	REACTOME_TRANSLATION	REACTOME_INFECTIOUS_DISEASE	REACTOME_RRNA_PROCESSING	REACTOME_CELL_CYCLE	REACTOME_SRP_DEPENDENT_COTRANSLATIONAL_PROTEIN_TARGETING_TO_MEMBRANE	REACTOME_EUKARYOTIC_TRANSLATION_INITIATION	Entrez	Ensembl	Gene Description
3303	HSPA1A	■	■	■	■		■							heat shock protein family A (Hsp70) member 1A [Source:HGNC Symbol;Acc:HGNC:5232]
6124	RPL4	■	■		■	■	■	■		■	■			ribosomal protein L4 [Source:HGNC Symbol;Acc:HGNC:10353]
6130	RPL7A	■	■		■	■	■	■		■	■			ribosomal protein L7a [Source:HGNC Symbol;Acc:HGNC:10364]
6137	RPL13	■	■		■	■	■	■		■	■			ribosomal protein L13 [Source:HGNC Symbol;Acc:HGNC:10303]
6181	RPLP2	■	■		■	■	■	■		■	■			ribosomal protein lateral stalk subunit P2 [Source:HGNC Symbol;Acc:HGNC:10377]
7175	TPR	■	■		■		■		■					translocated promoter region, nuclear basket protein [Source:HGNC Symbol;Acc:HGNC:12017]
7846	TUBA1A	■		■			■		■					tubulin alpha 1a [Source:HGNC Symbol;Acc:HGNC:20766]
2288	FKBP4	■		■										FKBP prolyl isomerase 4 [Source:HGNC Symbol;Acc:HGNC:3720]
3305	HSPA1L	■		■										heat shock protein family A (Hsp70) member 1 like [Source:HGNC Symbol;Acc:HGNC:5234]
10963	STIP1	■		■										stress induced phosphoprotein 1 [Source:HGNC Symbol;Acc:HGNC:11387]
8350	H3C1	■					■		■					H3 clustered histone 1 [Source:HGNC Symbol;Acc:HGNC:4766]
3020	H3-3A	■							■					H3.3 histone A [Source:HGNC Symbol;Acc:HGNC:4764]
8290	H3-4	■							■					H3.4 histone [Source:HGNC Symbol;Acc:HGNC:4778]
10111	RAD50	■							■					RAD50 double strand break repair protein [Source:HGNC Symbol;Acc:HGNC:9816]
22894	DIS3		■					■						DIS3 homolog, exosome endoribonuclease and 3'-5' exoribonuclease [Source:HGNC Symbol;Acc:HGNC:20604]
57050	UTP3		■					■						UTP3 small subunit processome component [Source:HGNC Symbol;Acc:HGNC:24477]
25929	GEMIN5		■											gem nuclear organelle associated protein 5 [Source:HGNC Symbol;Acc:HGNC:20043]
196513	DCP1B		■											decapping mRNA 1B [Source:HGNC Symbol;Acc:HGNC:24451]
84271	POLDIP3		■											DNA polymerase delta interacting protein 3 [Source:HGNC Symbol;Acc:HGNC:23782]
23020	SNRNP200		■											small nuclear ribonucleoprotein U5 subunit 200 [Source:HGNC Symbol;Acc:HGNC:30859]
6729	SRP54					■				■				signal recognition particle 54 [Source:HGNC Symbol;Acc:HGNC:11301]
9669	EIF5B					■					■			eukaryotic translation initiation factor 5B [Source:HGNC Symbol;Acc:HGNC:30793]
64976	MRPL40					■								mitochondrial ribosomal protein L40 [Source:HGNC Symbol;Acc:HGNC:14491]
5905	RANGAP1						■		■					Ran GTPase activating protein 1 [Source:HGNC Symbol;Acc:HGNC:9854]
11267	SNF8						■							SNF8 subunit of ESCRT-II [Source:HGNC Symbol;Acc:HGNC:17028]
11116	CEP43								■					centrosomal protein 43 [Source:HGNC Symbol;Acc:HGNC:17012]
51512	GTSE1								■					G2 and S-phase expressed 1 [Source:HGNC Symbol;Acc:HGNC:13698]
2956	MSH6													mutS homolog 6 [Source:HGNC Symbol;Acc:HGNC:7329]
2017	CTTN													cortactin [Source:HGNC Symbol;Acc:HGNC:3338]
57584	ARHGAP21													Rho GTPase activating protein 21 [Source:HGNC Symbol;Acc:HGNC:23725]
5160	PDHA1													pyruvate dehydrogenase E1 subunit alpha 1 [Source:HGNC Symbol;Acc:HGNC:8806]
5321	PLA2G4A													phospholipase A2 group IVA [Source:HGNC Symbol;Acc:HGNC:9035]
3920	LAMP2													lysosomal associated membrane protein 2 [Source:HGNC Symbol;Acc:HGNC:6501]
22927	HABP4													hyaluronan binding protein 4 [Source:HGNC Symbol;Acc:HGNC:17062]
6059	ABCE1													ATP binding cassette subfamily E member 1 [Source:HGNC Symbol;Acc:HGNC:69]
178	AGL													amylo-alpha-1, 6-glucosidase, 4-alpha-glucanotransferase [Source:HGNC Symbol;Acc:HGNC:321]
7317	UBA1													ubiquitin like modifier activating enzyme 1 [Source:HGNC Symbol;Acc:HGNC:12469]
3069	HDLBP													high density lipoprotein binding protein [Source:HGNC Symbol;Acc:HGNC:4857]
11331	PHB2													prohibitin 2 [Source:HGNC Symbol;Acc:HGNC:30306]
3925	STMN1													stathmin 1 [Source:HGNC Symbol;Acc:HGNC:6510]
4522	MTHFD1													methylenetetrahydrofolate dehydrogenase, cyclohydrolase and formyltetrahydrofolate synthetase 1 [Source:HGNC Symbol;Acc:HGNC:7432]
10061	ABCF2													ATP binding cassette subfamily F member 2 [Source:HGNC Symbol;Acc:HGNC:71]
10801	SEPTIN9													septin 9 [Source:HGNC Symbol;Acc:HGNC:7323]
11075	STMN2													stathmin 2 [Source:HGNC Symbol;Acc:HGNC:10577]
113146	AHNAK2													AHNAK nucleoprotein 2 [Source:HGNC Symbol;Acc:HGNC:20125]
23148	NACAD													NAC alpha domain containing [Source:HGNC Symbol;Acc:HGNC:22196]
253943	YTHDF3													YTH N6-methyladenosine RNA binding protein 3 [Source:HGNC Symbol;Acc:HGNC:26465]
26586	CKAP2													cytoskeleton associated protein 2 [Source:HGNC Symbol;Acc:HGNC:1990]
282973	JAKMIP3													Janus kinase and microtubule interacting protein 3 [Source:HGNC Symbol;Acc:HGNC:23523]
29889	GNL2													G protein nucleolar 2 [Source:HGNC Symbol;Acc:HGNC:29925]
4678	NASP													nuclear autoantigenic sperm protein [Source:HGNC Symbol;Acc:HGNC:7644]
49855	SCAPER													S-phase cyclin A associated protein in the ER [Source:HGNC Symbol;Acc:HGNC:13081]
55573	CDV3													CDV3 homolog [Source:HGNC Symbol;Acc:HGNC:26928]
57062	DDX24													DEAD-box helicase 24 [Source:HGNC Symbol;Acc:HGNC:13266]
83447	SLC25A31													solute carrier family 25 member 31 [Source:HGNC Symbol;Acc:HGNC:25319]
8458	TTF2													transcription termination factor 2 [Source:HGNC Symbol;Acc:HGNC:12398]
9908	G3BP2													G3BP stress granule assembly factor 2 [Source:HGNC Symbol;Acc:HGNC:30291]
